# Discrimination, Stress, and Health Across the Life Course

**DOI:** 10.1093/geroni/igab046.717

**Published:** 2021-12-17

**Authors:** Roland Thorpe, Carl V Hill

## Abstract

There is a paucity of research that seeks to understand why race disparities in health across the life course remain elusive. Two such explanations that have been garnering attention is stress and discrimination. This symposium contains papers seeking to address the impact of discrimination or stress on African American health or health disparities across the life course. Brown and colleagues examine the differential effects of chronic stress exposure by means of latent class analysis on mental and physical health in the HRS. Analysis revealed four subgroups, each demonstrated a typological response pattern with the most pronounced health consequences for high stress exposure, appraisal and few or no coping mechanisms. This suggests an alternative approach to examining the stress-health link by using a combined person- and variable-centered approach. Thomas Tobin and colleagues evaluate the life course processes through which early life racial discrimination (ELRD) and racial centrality shape adult allostatic load (AL) among older Blacks in the Nashville Stress and Health Study. Findings indicate that racial centrality is protective against adult high AL for those who experienced racial discrimination as children or adolescents. Cobb and colleagues examine how multiple attributed reasons for everyday discrimination relates to all-cause mortality risk among older Blacks in HRS. The authors report the 3 or more attributed reasons for everyday discrimination is a particularly salient risk factor for mortality in later life. This collection of papers provides insights into how discrimination or stress impacts African American health or health disparities in middle to late life.

